# Vitrectomy with complete posterior hyaloid removal for ischemic central retinal vein occlusion: Series of cases

**DOI:** 10.1186/1471-2415-5-10

**Published:** 2005-05-20

**Authors:** Carlos Leizaola-Fernández, Luis Suárez-Tatá, Hugo Quiroz-Mercado, Juner Colina-Luquez, J Fromow-Guerra, Juan M Jiménez-Sierra, Jose L Guerrero-Naranjo, Virgilio Morales-Cantón

**Affiliations:** 1 Department of Retina. Hospital "Dr. Luis Sánchez Bulnes" Asociación Para Evitar la Ceguera en México (APEC), Distrito Federal, México; 2 Department of Electrophisiology. Hospital "Dr. Luis Sánchez Bulnes" Asociación Para Evitar la Ceguera en México (APEC), Distrito Federal, México

## Abstract

**Background:**

Central retinal vein occlusion (CRVO) is a common retinal vascular disorder with potentially complications: (1) persistent macular edema and (2) neovascular glaucoma. No safe treatment exists that promotes the return of lost vision. Eyes with CRVO may be predisposed to vitreous degeneration. It has been suggested that if the vitreous remains attached to the macula owing to a firm vitreomacular adhesion, the resultant vitreous traction can cause inflammation with retinal capillary dilation, leakage and subsequent edema6. The roll of vitrectomy in ischemic CRVO surgical procedures has not been evaluated.

**Case presentation:**

This is a non comparative, prospective, longitudinal, experimental and descriptive series of cases. Ten eyes with ischemic CRVO. Vitrectomy with complete posterior hyaloid removal was performed. VA, rubeosis, intraocular pressure (IOP), and macular edema were evaluated clinically. Multifocal ERG (m-ERG), fluorescein angiography (FAG) and optic coherence tomography (OCT) were performed. Follow-up was at least 6 months. Moderate improvement of visual acuity was observed in 60% eyes and stabilized in 40%. IOP changed from 15.7 ± 3.05 mmHg to 14.9 ± 2.69 mmHg post-operative and macular edema from 976 ± 196 μm to 640 ± 191 μm to six month. The P1 wave amplitude changed from 25.46 ± 12.4 mV to 20.54 ± 11.2 mV.

**Conclusion:**

A solo PPV with posterior hyaloid removal may help to improve anatomic and functional retina conditions in some cases. These results should be considered when analyzing other surgical maneuvers.

## Background

Central retinal vein occlusion (CRVO) is a common retinal vascular disorder with potentially complications like reduced vision resulting from extensive intraretinal hemorrhage, retinal ischemia and persistent macular edema and neovascular glaucoma secondary to iris neovascularization. Severe visual loss may develop, including blindness. Capillary nonperfusion and retinal ischemia develop in 34% of patients with CRVO. Iris neovascularization and neovascular glaucoma may occur in 45–85% of ischemic CRVO [15,20,23,24]. No safe treatment exists that promotes the return of lost vision. Treatments that target the secondary effects of venous occlusion, such as grid laser photocoagulation for macular edema and prophylactic panretinal laser photocoagulation for nonperfused CRVO, were shown to be ineffective in improving visual acuity in the Central Vein Occlusion Study (CVOS) [21,22]. Attempts to bypass venous obstruction in CRVO using laser chorioretinal anastomosis have been performed with limited success and significant complications [3,11]. Radial optic neurotomy has been shown to be beneficial for the treatment of CRVO [13]. Most patients who develop CRVO (50 – 70%) have associated hypertension, cardiovascular disease, or diabetes mellitus [5]. The treatment of these systemic conditions is not effective in the management of the ocular complications. It has been postulated that vitreous traction may play a role in the pathogenesis of cystoid macular edema [16,17]. Eyes with CRVO may be predisposed to vitreous degeneration. It has been suggested that if the vitreous remains attached to the macula owing to a firm vitreomacular adhesion, the resultant vitreous traction can cause inflammation with retinal capillary dilation, leakage and subsequent edema [16]. Pars plana vitrectomy is believed to eliminate this mechanical traction and increase oxygenation to the retina and to reduce the risk of macular edema and neovascularization. The purpose of this study was to perform an assessment of the safety and efficacy of pars plana vitrectomy combined with complete posterior hyaloid removal in ischemic CRVO.

### Case presentation

Non comparative, prospective, longitudinal, experimental and descriptive series of cases of patients with ischemic CRVO referred to Hospital Luis Sanchez Bulnes, Asociación para Evitar la Ceguera en México, México City. The study was approved by Hospital Review Committee The inclusion criteria were: confirmed presence of central retinal vein occlusion, less than 6 months evolution with visual acuity (VA) of <20/100 or those whose VA decreased more than 50% during the follow-up, an over 10 disc diameter area of nonperfused retina, intraocular pressure ≤ 21 mmHg, age ≥ 21 years old, afferent pupillary defect, ability to obtain good quality fundus photographs and angiograms and absence of neovascularization. The exclusion criteria in the study eye were: intercurrent eye disease that is likely to affect VA over study period, presence of any diabetic retinopathy, other retinal vascular disease, vitreous hemorrhage, presence of neovascularization (iris, angle, retina and disk) or previous laser treatment. Best corrected ETDRS visual acuity (BCVA), relative afferent pupillary defect (RAPD), slit-lamp examination, indirect ophthalmoscopy, fundus photography, fluorescein angiography (FA), multifocal electroretino*graphy (mERG) and optic coherence tomography (OCT) were performed preoperatively and at 1, 3, and 6 months postoperatively. Follow-up was at least 6 months.

### Surgical procedure

After informed consent was obtained and a full explanation of the risks and benefits and the experimental nature of this procedure were conveyed to the patient (in accordance with the Helsinki declaration), patients underwent a standard three port pars plana vitrectomy was performed, during which the posterior hyaloid was removed using active aspiration. The sclerostomy sites and conjunctiva were closed in the usual fashion. No panretinal photocoagulation and gas tamponade were used.

### OCT and mERG

Macular thickness was analyzed with OCT (Humphrey 1000; Humphrey Instruments, San Leandro, California). Measurement of central macular thickness was evaluated according to Hee protocol [7], which in each eye, six consecutive tomographic cuts were obtained in a radial pattern, surrounding the fovea at similar angular distances among them. Each one of these cuts was oriented along a line that intersects the foveal center. The central foveal thickness was registered at the intersection of the 6 cuts.

The electrical function of the macular area was determined by multifocal electroretinography (mERG). The RETI-scan™ multifocal system, produced by Roland Consult, was used for this purpose. The stimulation and recording of the mERG were performed using the m-sequence technique. Contact lens ERG-JET electrodes as well as one ground electrode in the center of forehead and two reference electrodes in the temporal region of the patient were placed. The stimulus, consisting of 61 hexagons covering a visual field of 30°, was presented on a monitor (ELSA 20"-VGA-monitor) with a frame rate of 75 Hz at a distance of 28 cm from the patient's eye. Each element alternated between black and white (93% contrast, mean luminance 51.8 cd/m^2^). The patient was instructed to maintain fixation on longitudinal axes intersecting one focal point. The amplifier setting was 100 μV; the lower cut off frequency was 10 Hz and the upper cut off frequency was 100 Hz. No notch filter was used. Each recording session was subdivided into 8 recording segments of approximately 47 seconds. The signals were registered with sampling intervals of 83 mseg. The results were distributed in 5 concentric rings obtaining for each ring, the N1 amplitude, P1 amplitude, and the implicit times of N1 and P1. The amplitude of the positive component P1 was measured, and the response density (nV/deg^2^) was calculated by dividing the response amplitude (nV) by the retinal area (deg^2^).

## Results

The study data are displayed in Table 1 and 2. Patient's age ranged from 57 to 74 years, with a mean of 65.13 years. There were 4 females and 6 males. All patients were Hispanic. Initial treatment was attempted between January 2002 and February 2003. The time interval range from diagnosis to treatment was 37 to 270 days, with a mean of 103.13 days. In all patients, the post treatment follow-up examination was at least 6 months. Four patients had diabetes mellitus type 2 and six patients had systemic arterial hypertension.

The median preoperative BCVA was 20/600 (range, 20/1600 to 20/300). The median postoperative BCVA was 20/300 (range, 20/1600 to 20/100). VA was unchanged in 4 of 10 eyes (40%) and improved in 6 of 10 eyes (60%). None eye had no light perception. The median preoperative PIO was 15.7 ± 3.05 mmHg and the median postoperative PIO was 14.9 ± 2.68 mmHg. Central macular edema registered by OCT changed from 976 ± 196 μm to 640 ± 191 μm to six month. The P1 wave amplitude of the central ring varied from 25.46 ± 12.4 mV to 20.54 ± 11.2 mV to final follow up.

None eye suffered retinal o disc neovascular membrane. The iris neovascularization was presented in 3 eyes (30%). All these eyes were treated with extensive photocoagulation. None of these eyes suffered neovascular glaucoma. Eight eyes presented middle opacity of lens.

## Discussion

Fine and Brucker [4] and Wallow and 3 colleagues [25] demonstrated that cystoid macular edema began with ischemia and intracytoplasmic swelling of Müller cells, and speculated that an intrinsic metabolic agent could cause these changes. The vitreous is believed to store pharmacologic agents that may cause macular edema. Hikichi and 2 colleagues[8] proposed that in eyes with vitreous macular attachment, the macular edema is persistent because those metabolic agents remain in contact with the macula. Also they observed that a complete posterior vitreous detachment seems to protect against retinal or optic disc neovascularization. Another theory asserts that because vitreous fibres extending from the vitreous base to the macula and Müller cell attachment to the internal limiting lamina are prominent in this region [17,20] centripetal traction caused by vitreous contraction after CRVO and transmitted to Müller cells by the vitreous fibres attached to the macula may cause swelling of these cells. Kado and 6 colleagues [9] showed that macular edema lasted longer in eyes with vitreous macular attachment than in those whose vitreous was detachment from the macula, due to centripetal traction transmitted to the Müller cells by vitreous fibres inserted into the macula. Based on these facts, we proposed that a vitrectomy could eliminate the metabolic agents present in the vitreous as well as the mechanical traction, and in this way might protect against retinal and optic disc neovascularization. Also, pars plana vitrectomy may beneficial effects upon retinal ischemic by allowing circulation in the vitreous cavity of fluid oxygenated by unaffected retina or others sites in the eye such as the ciliary body [10,19]. In our patients, a standard three port pars plana vitrectomy was performed followed by creation of posterior hyaloid separation and removal of the posterior cortical vitreous. The central macular edema registered by OCT changed from 976 ± 196 μm pre operative to 640 ± 191 μm to six month. All patients presented persistent cystoid macular edema to six month.

The level of visual improvement seems relatively good when compared with the results of conventional treatment such as retinal photocoagulation, or only observation. Although 40% of eyes maintained stable visual acuity and 60% of eyes had visual improvement, there was still considerable long-term visual morbidity in this series. Only two eyes had final VA better than 20/200 (20/100 in both eyes). The rest of eyes had a final VA of 20/200 or less. Long-term visual morbidity may be corresponding to residual perfused cystoid macular edema or to degenerative squealer of previous chronic edema. None patient presented significant complication post treatment such as neovascular glaucoma or retinal detachment. Most patients with CRVO lose their visual acuity permanently because they develop macular edema or ischemia. The degree of retinal hypoxia and infarction and probably the extent of abnormal vascular permeability may be are reflected in electroretinogram (ERG) amplitude and timing changes. In our patients, the P1 wave amplitude changed from 25.46 ± 12.4 mV pre operative to 20.54 ± 11.2 mV to six month. Once the VA of these patients is severely reduced, 80% of them cannot expect any improvement spontaneously [2,14,23]. In this study, the pre treatment visual acuity in all eyes ranged from 20/800 to 20/300 (20/300). It is recognized that earlier intervention probably is favourable to later to prevent macular scarring from longstanding edema [12,18]. In our series, in eyes with early treatment (≤90 days), 4 eyes (67%) improved VA and 2 eyes (33%) were unchanged. In eyes with later treatment (>90 days), 2 eyes (50%) improved VA and 2 eyes (50%) were unchanged.

In severe cases of ischemic CRVO, the role of the vitreous was not observed. Increased venous pressure, which leads to damage of the capillary endothelium and breakdown of the blood-retinal barrier, was reported to be the main cause of macular edema in CRVO [6,25]. Because capillary damage may be aggravated sufficiently to negate the effect of complete posterior vitreous detachment on macular edema resolution in severe cases, the vitreoretinal relationship can only influence the prognosis of macular conditions when there is mild capillary damage in the macular region.

In the ischemic type of CRVO, the reported prevalence of posterior segment and iris neovascularization ranges from 0% to 55% and from 16% to 67%, respectively [15,20,23,24]. In our series, none eyes presented retinal or optic neovascularization membrane. The iris neovascularization was presented in 3 patients (30%). The posterior vitreous detachment seems to play a protective role against retinal or optic disc neovascularization in central retinal vein occlusion [1,8], although the vitreoretinal relationship may not influence the development of iris neovascularization.

The efficacy and safety of vitrectomy in these patients is still in question. Further studies such as a randomized controlled clinical trial are needed to determine whether vitrectomy is of benefit in ischaemic CRVO. Our results suggest that a solo PPV with posterior hyaloid removal may help to improve anatomic and functional retina conditions in some cases. These results should be considered when analyzing other surgical maneuvers.

## Conclusion

A solo PPV with posterior hyaloid removal may help to improve anatomic and functional retina conditions in some cases. These results should be considered when analyzing other surgical maneuvers.

## Competing interests

The author(s) declare that they have no competing interests.

## Authors' contributions

CLF: participated in the sequence alignment and drafted the manuscript

LST: participated in the sequence alignment and drafted the manuscript

HQM: participated in the sequence alignment

JCL: participated in the sequence alignment, sending the manuscript and doing the corrections

JFG: participated in the design of the study and performed the statistical analysis

JMJS: participated in doing and analysis of electrophysiological studies

JLGN participated in doing all surgical procedures

VMC: participated in its design and coordination and helped to draft the manuscript

## Pre-publication history

The pre-publication history for this paper can be accessed here:



## Figures and Tables

**Table 1:** Patients Characteristics

Case	Age (years)	Sex	HBP	DM	Duration (days)*	VA initial	VA 3 month	VA final	Follow-Up (month)
1	57	M	N	N	120	20/1600	20/1600	20/1600	12
2	66	F	N	Y	66	20/1600	20/1600	20/800	10
3	72	M	N	Y	75	20/1600	20/1600	20/1600	8
4	70	M	Y	N	80	20/1600	20/1600	20/1600	8
5	59	M	N	Y	37	20/300	20/100	20/100	7
6	74	F	Y	Y	98	20/800	20/100	20/100	7
7	65	M	Y	N	65	20/300	20/300	20/200	6
8	58	M	N	N	80	20/1600	20/800	20/600	6
9	64	F	Y	N	180	20/1600	20/1600	20/1600	6
10	60	F	Y	N	90	20/600	20/300	20/300	6

HBP: high blood pressure; DM: diabetes mellitus; VA: visual acuity; IOP: intraocular pressure; M: masculine; F: feminine; Y: yes; N: no

* Time interval range from diagnosis to treatment

**Table 2:** IOP, macular thickness and mERG values

	IOP mmHg		Macular thickness (μm) OCT		P1 wave amplitude (mV) mERG	
Case	Preop	Postop	Preop	Postop	Preop	Postop
1	12	14	1100	800	44.2	16.4
2	16	18	1176	800	13.8	12.3
3	12	11	1200	750	7.0	30.2
4	18	16	1000	375	23.0	6.1
5	14	18	920	477	38.0	40.0
6	12	10	780	320	9.0	24.9
7	15	16	1000	700	23.8	14.7
8	20	18	1300	750	17.7	6.01
9	18	14	730	650	36.7	29.2
10	18	16	850	700	25.0	30.5

IOP: intraocular pressure; OCT: optic coherence tomography; mERG: multifocal electroretinogram; Preop: preoperative; Postop: postoperative
